# Application of Locked Nucleic Acid (LNA) Oligonucleotide–PCR Clamping Technique to Selectively PCR Amplify the SSU rRNA Genes of Bacteria in Investigating the Plant-Associated Community Structures

**DOI:** 10.1264/jsme2.ME14061

**Published:** 2014-07-17

**Authors:** Makoto Ikenaga, Masao Sakai

**Affiliations:** 1 Faculty of Agriculture, Kagoshima University, 1–21–24, Korimoto, Kagoshima 890–0065, Japan

**Keywords:** plant organelle, bacteria, community structure, SSU rRNA gene, locked nucleic acid (LNA) oligonucleotide–PCR clamping

## Abstract

The simultaneous extraction of plant organelle (mitochondria and plastid) genes during the DNA extraction step is a major limitation in investigating the community structures of bacteria associated with plants because organelle SSU rRNA genes are easily amplified by PCR using primer sets that are specific to bacteria. To inhibit the amplification of organelle genes, the locked nucleic acid (LNA) oligonucleotide–PCR clamping technique was applied to selectively amplify bacterial SSU rRNA genes by PCR. LNA oligonucleotides, the sequences of which were complementary to mitochondria and plastid genes, were designed by overlapping a few bases with the annealing position of the bacterial primer and converting DNA bases into LNA bases specific to mitochondria and plastids at the shifted region from the 3′ end of the primer-binding position. PCR with LNA oligonucleotides selectively amplified the bacterial genes while inhibited that of organelle genes. Denaturing gradient gel electrophoresis (DGGE) analysis revealed that conventional amplification without LNA oligonucleotides predominantly generated DGGE bands from mitochondria and plastid genes with few bacterial bands. In contrast, additional bacterial bands were detected in DGGE patterns, the amplicons of which were prepared using LNA oligonucleotides. These results indicated that the detection of bacterial genes had been screened by the excessive amplification of the organelle genes. Sequencing of the bands newly detected by using LNA oligonucleotides revealed that their similarity to the known isolated bacteria was low, suggesting the potential to detect novel bacteria. Thus, application of the LNA oligonucleotide–PCR clamping technique was considered effective for the selective amplification of bacterial genes from extracted DNA containing plant organelle genes.

Plants represent complex, as well as spatially and temporally diverse ecological habitats to bacteria. Bacteria that associate with plants can have beneficial, neutral, or deleterious effects on plant growth (23). Beneficial bacteria have been shown to promote plant growth or protect plants from diseases (29), while deleterious bacteria inhibit growth (32). It is important to control the optimum constitution of plant-associated bacteria in order to maintain plant health. Therefore, many efforts have been made to characterize community structures.

The use of traditional culture-dependent techniques generally results in diversity assessments between 0.001% and 15% of the total bacterial population (1). Since this phenomenon is also applicable to bacteria associated with plants (7), most of the population of plant-associated microbial community still remains unclear. In contrast, culture-independent molecular techniques are useful tools for studying bacterial communities in ecosystems because they provide information on the differences between dominant community structures, including non-cultured and as yet unexploited bacteria. This approach has enabled comparisons of the structures of a previously cultivated fraction of a bacterial community (22).

Although culture-independent molecular techniques are beneficial, they also create a major limitation when investigating the community structures of plant-associated bacteria because of the high contamination of plant organelle (mitochondria and plastid) genes (6, 12, 13, 27, 49). This phenomenon is due to the primer set that is used to amplify bacterial SSU rRNA genes, which is also homologous to mitochondria and plastid SSU rRNA genes, resulting in a high abundance of plant organelle ribosomal sequences in the PCR products. Therefore, this undesirable amplification has led to the underestimation of community structures and delayed understanding of the effects of bacteria on plant growth. A more detailed insight into the bacteria community will lead to the development of agricultural applications as a microbial resource by isolating bacteria using culture-dependent techniques based on the information obtained by culture-independent molecular techniques.

In response to this critical limitation, Sakai and Ikenaga (39) used the peptide nucleic acid (PNA)-PCR clamping technique to investigate the community structures of rhizobacteria associated with plant roots. PNA-PCR clamping specifically inhibits the amplification of mitochondria and plastid SSU rRNA genes using PNA oligomers, which are perfectly homologous to the organelle sequences and inoperative as primers during PCR cycles, while allowing the amplification of bacterial genes. Thus, the application of the PNA-PCR clamping technique was considered effective for the selective amplification of bacterial SSU rRNA genes when the extracted DNA contained plant organelle genes. However, this approach was insufficient because PNA oligomers could only be designed in the binding region of the 1492r reverse primer due to the following severe restriction rules; 1) the size must be 18 mers or less, 2) purine base (A and G) content must be 60% or less in the oligomer, 3) stretches of 5 and more Gs or 6 and more purine bases must be avoided (http://www.fasmac.co.jp/bio/pna/aboutPNA.html).

Locked nucleic acid (LNA) is an artificial nucleotide analogue that contains a methylene bridge connecting the 2′-oxygen of ribose with the 4′-carbon (18, 35). This bridge has been shown to result in a locked 3′-*endo* conformation with reduced conformational flexibility (19, 24). The LNA base can be incorporated into DNA oligonucleotide sequences (3, 17). Therefore, the LNA oligonucleotide possesses extraordinary mismatch sensitivity to complementary nucleic acids in LNA/DNA hybrids (41, 48) and higher thermal stability than the DNA oligonucleotide (18, 20). The LNA oligonucleotide also has the following advantages: 1) it can be designed similar to a normal primer while avoiding stretches of 4 and more LNA bases (https://www.exiqon.com/oligo-tools); 2) it is inoperative as a primer upon phosphorylation of the 3′ end; and 3) it is designable by overlapping the primer-binding position with the forward primer side. Accordingly, the LNA oligonucleotide–PCR clamping technique is considered to be applicable at both the forward and reverse primer-binding regions.

In the present study, we first designed LNA oligonucleotides specific for mitochondria and plastid SSU rRNA genes, then examined their effective concentrations for the selective amplification of bacterial SSU rRNA genes by PCR. Secondary we employed PCR-DGGE and sequencing analyses (14, 46) to compare community structures by observing the band patterns generated with and without LNA oligonucleotides, and discussed the effects of the LNA oligonucleotide– PCR clamping technique to solve the critical drawback in investigating the community structures of plant-associated bacteria.

## Materials and Methods

### Design of LNA oligonucleotides

The SSU rRNA gene sequences of plant organelles (mitochondria and plastid) and bacteria were obtained from the DNA Data Bank of Japan (DDBJ, http://www.ddbj.nig.ac.jp/index-e.html). The Ribosomal Data Project Release 11 (RDP11, http://rdp.cme.msu.edu/index.jsp) was also used to collect bacterial and plastid genes. Bacterial genes were widely collected from 29 phyla and 6 candidate divisions with consideration of phylogenetic diversity. The sequences of type strains were used for alignments. The SSU rRNA gene sequences were aligned together with a bacterial primer using CLUSTAL W version 1.7 (45) in order to find the specific sequences for plant organelles at a region that shifted several nucleotides from the 3′ end of the annealing position of the bacterial primer, while bacterial sequences were highly divergent at the corresponding region. The sequences of mitochondria and plastids collected are listed in supplementary Table S1 and S2, respectively.

After alignment with the primer, the LNA oligonucleotides were designed by overlapping a few DNA bases with the annealing position of the bacterial primer at the extension side and converting the DNA bases, which were only specific for plant organelles, into LNA bases at the shifted region. The 3′ end of the LNA oligonucleotides was phosphorylated to avoid extension during PCR. The sequences of the designed LNA oligonucleotides were then analyzed using the software ROSE version 1.1.3 (2) and Probe Match included in RDP 11 to ascertain whether identical sequences existed in the bacterial SSU rRNA genes.

### Preparation of plant samples for DNA extraction

Rice (*Oryza sativa* cv. Koshihikari) and wheat (*Triticum aestivum* cv. Chikugoizumi) were cultivated in upland conditions. Soil was collected from an agricultural field at Kagoshima University (latitude +31° 34′ 25″, longitude +130° 32′ 34″) and sieved through a 4-mm mesh. Three kg of soils were put into 1/5,000a size pots after having mixed 6.0 g of (NH_4_)_2_SO_4_, 6.0 g of Ca_3_(PO_4_)_2_, and 2.4 g of KCl. Seeds were moistened in Petri dishes until germination and placed in the potted soil. After 3 weeks of cultivation, the plants were harvested, and the leaves and roots were separately collected. The samples were washed in 10 mM phosphate buffer (pH 7.0) to remove attachments including soil particles, cut into millimeter sizes, and ground with a sterilized mortar and pestle. Rice and wheat seeds were also washed in phosphate buffer and ground.

To prepare aseptic roots, the seed hulls of rice and wheat were removed and treated with 1% NaOCl (v/v) for 60 min to sterilize microorganisms. The seeds were then rinsed six times in sterile distilled water and placed on 1/10-strength tryptic soy agar (28). The seeds were incubated at 30°C for 6 d until the radicles grew to 15–20 mm in length. The roots, which were confirmed to be aseptic because no microorganism colonies formed beside or around the roots on the agar media, were collected and ground.

### DNA extraction from plant samples

Approximately 0.4 g (fresh weight) of ground leaves, roots, and aseptic roots and 0.2 g (fresh weight) of ground seeds were used in duplicate for DNA extraction using the FastDNA SPIN Kit for Soil (MP Biomedicals, Solon OH, USA), according to the manufacturer’s instructions. Controls without samples were also prepared to evaluate the potential of DNA contamination from the reagents. Extracted DNA was stored at −20°C until PCR amplification.

### Determination of the effective annealing temperature for LNA oligonucleotides

To determine the effective annealing temperature for the LNA oligonucleotides, PCR amplification was performed at different annealing temperatures for the bacterial primer by using a PCR thermal cycler (PC350 ASTEC, Fukuoka, Japan). DNA extracted from rice and wheat roots was used for the amplification. The PCR mixture contained 12.5 μL of *Premix* Hot Start Version (Takara, Otsu, Japan), 1.0 μL of each primer (20 pmol μL^−1^), and 1.0 μL of the DNA template. Sterilized ultrapure water was added to a total volume of 25 μL. The amplification conditions were as follows: 94°C for 3 min (initial denaturation), followed by 30 cycles at 94°C for 1 min, annealing from 60°C to 72°C (2°C increments) for 1 min, and 72°C for 2 min, with a final extension step at 72°C for 10 min. After the amplification, aliquots of the PCR products were electrophoresed on a 1.5% agarose gel, and the effective annealing temperature of the LNA oligonucleotides was visually estimated from the product intensities.

### Determination of effective concentration of LNA oligonucleotides

The effective concentration of the LNA oligonucleotides was determined. The extracted DNAs were used in the amplification together with the LNA oligonucleotides. The PCR mixtures were prepared to contain serial concentrations of LNA oligonucleotides of 0 μM, 0.5 μM, 1.0 μM, 2.0 μM, 3.0 μM, and 4.0 μM. PCR tubes including the DNA extracted from aseptic roots were also prepared without adding the LNA oligonucleotide as the control.

In the LNA oligonucleotide–PCR clamping technique, the annealing step of the LNA oligonucleotides needed be inserted between the denaturation step and annealing step for the bacterial primer. The incubation period of 1 min was inserted between the steps. After the amplification, aliquots of the PCR products were electrophoresed on 1.5% agarose gel, and the effective concentration of the LNA oligonucleotides was estimated from the electrophoresis results.

### Nested PCR amplification

The PCR products obtained using the effective annealing temperature and concentration of LNA oligonucleotides were again amplified using the bacterial primer for DGGE and sequencing analyses. Prior to nested PCR, the PCR products were purified using the High Pure PCR Product Purification Kit (Roche, Indianapolis IN, USA). The purified products were then serially diluted and used for amplification.

Nested PCR amplification was also performed for the PCR products amplified without LNA oligonucleotides in order to compare DGGE patterns. In addition, the DNA extracted from aseptic roots was directly amplified using the bacterial primer employed for DGGE analysis with the same PCR conditions. The products were used to determine the mobility positions of mitochondria and plastid genes in the DGGE gel.

### DGGE and sequencing analyses

DGGE was performed in 8% (w/v) acrylamide gel containing a linear chemical gradient ranging from 30% to 60% denaturant (100% denaturant, 7 M urea and 40% [v/v] formamide) (30). Amplicons of approximately 600 ng were loaded onto the DGGE gel and electrophoresed at 60°C and 100 V for 14 h using a DCode universal mutation detection system (BioRad Laboratories, Hercules CA, USA). After electrophoresis, the gel was stained with SYBR Gold (Life Technologies Japan, Tokyo, Japan) for 30 min and photographed under UV illumination.

The DGGE bands, which were newly detected using LNA oligonucleotides, were excised from the gel and directly amplified as the DNA template. A mobility check of the amplified band was performed to confirm whether the position of the band was the same as that of the original by replicating the DGGE analysis under identical conditions.

Cycle sequencing was conducted for the isolated DGGE bands using the BigDye Terminator v3.1 Cycle Sequencing kit (Life Technologies Japan), according to the manufacturer’s instructions. DNA sequencing was performed using the ABI 3500*xL* Genetic Analyzer (Life Technologies Japan). The reverse primer used for nested PCR was used also for sequencing.

### Nucleotide sequence accession number

The sequences obtained in this study are available on the DNA databases under the accession numbers from AB908169 to AB908176.

## Results and Discussion

### Selection of the forward primer

The forward primers 27f (37), 41f (9), 63f (26), 341f (30), 519f (25), and 799f (4) were examined individually to find the designable region of the LNA oligonucleotides specific for mitochondria and plastids. All the primers were used for PCR amplification of the bacterial SSU rRNA genes in the community analyses. Regarding the reviver primer, 1492r was used to design the LNA oligonucleotides specific for mitochondria and plastids because a designable sequence was previously shown to exist at the shifted region from the 3′ end of the primer-binding site (39). Other reverse primers such as 517r (30), 783r (38), 907r (15), 1401r (34), and 1525r (10) contained no designable sequences at the corresponding regions.

As a result of the sequence alignment with a primer selected from the examined forward primers, the designable region of the LNA oligonucleotide was only found in the 63f primer-binding site. However, according to the ROSE version 1.1.3, coverage of the 63f primer, the sequence of which was 5′-CAGGCCTAACACATGCAAGTC-3′, was 14.8% (2). The bacterial primers used for community analyses generally have the following coverages: 27f, 72.9%; 341f, 86.8%; 799f, 82.6%; 517r, 84.0%; 907r, 86.2%; and 1492r, 81.2% as calculated with the ROSE software. To increase the coverage of the 63f primer, six DNA bases were degenerated and a modified 63f primer was designed. Coverage was increased to 76.3%, and the sequence was 5′-YRKGCYT WAYACATGCAAGTC-3′.

### Determination of an effective annealing temperature for LNA oligonucleotides

**Fig. 1:**
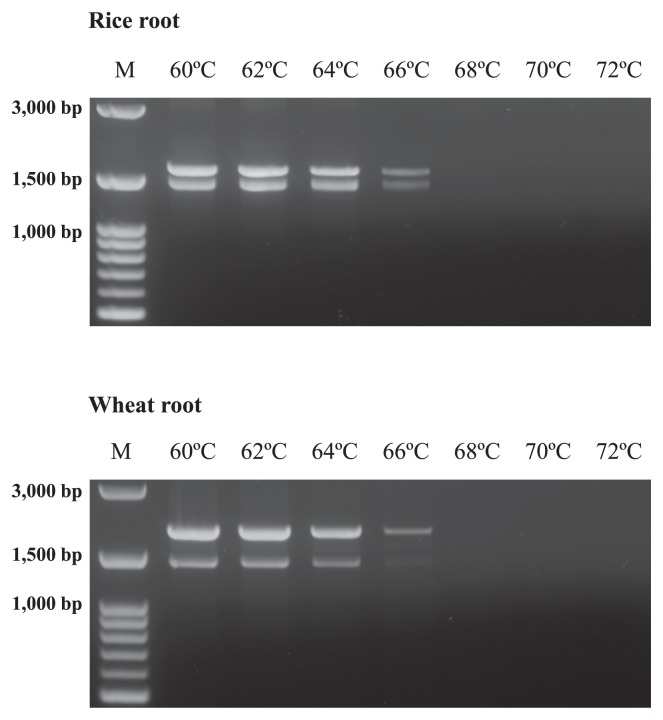
Agarose gel electrophoresis of the PCR products (modified 63f and 1492r) derived from rice and wheat roots to determine an effective annealing temperature for LNA oligonucleotides. The annealing temperature ranged from 60°C to 72°C, with increments of 2°C. “M” is the marker of 100 bp ladders.

In the LNA oligonucleotide–PCR clamping technique, the annealing step of the LNA oligonucleotides needed to be inserted between the denaturation step and annealing step of the bacterial primer in order to inhibit the primer from annealing to the plant organelle SSU rRNA genes (44, 47). In addition, the annealing temperature of the LNA oligonucleotides needed to be higher than that of the bacterial primers, such that the bacterial primers were inoperative at this temperature to avoid annealing of the bacterial primers to the plant organelle genes during PCR. If the bacterial primers could be annealed at these high annealing temperatures, plant organelle genes were amplified while decreasing the effects of PCR clamping by LNA oligonucleotides. To avoid annealing of the bacterial primers during the annealing step of the LNA oligonucleotide, PCR was performed with bacterial primers in order to determine the effective annealing temperature for the LNA oligonucleotides (Fig. 1). This experiment was performed to examine the temperature that bacterial primers were inoperative and to utilize this temperature to anneal LNA oligonucleotides because thermal stability was increased when DNA bases were converted to LNA bases.

As shown in Fig. 1, the amplification products of DNA extracted from rice and wheat roots were detected with high intensities up to 64°C. However, these intensities decreased at 66°C, and no products were detected at 68°C, 70°C, and 72°C. This result indicated that the bacterial primer set was operative up to 66°C. However, 66°C is considered to be a relatively high annealing temperature because the melting temperatures (*Tm*) of the modified 63f and 1492r primers were 61.0°C and 55.0°C, respectively. Therefore, a high temperature resulted in low product intensities after the PCR cycles. Based on these results, the *Tm* value of the LNA oligonucleotides was set to 70°C, and the annealing step of the LNA oligonucleotides was performed at 70°C to promote the effects of PCR clamping.

### Design of LNA oligonucleotides

The alignment data of the representative sequences of plant organelle and bacterial genes together with a primer and the designed LNA oligonucleotide are shown in Table 1. As shown in Tables 1A and 1B, the LNA oligonucleotides specific for mitochondria genes were designed to overlap with a few nucleotides with modified 63f (Table 1A) and 1492r (Table 1B) primers-binding sites. The sequences of the LNA oligonucleotides were identical to those of the mitochondria genes in almost all plants, whereas a few exceptions were observed to have one or two mismatches (see Supplementary Table S1 for details). The LNA oligonucleotides that competed with modified 63f and 1492r were named LNA-Mit63 and LNA-Mit1492, respectively.

In contrast, the plastid sequences at the shifted region were generally distinguished into three groups (Table 1C for modified 63f and Table 1D for 1492r). First group included the major crops such as rice, wheat, maize, and sugarcane, which belonged to the family *Poaceae*. The second group included different major crops such as soybean, carrot, cassava, spinach, sugar beet cucumber, tobacco, and cotton. The third group exhibited the hetero-sequence; the first group sequence for the forward primer site and second group sequence for the reverse primer site (see Supplementary Table S2 for details). Since rice and wheat are the most important crops worldwide, we designed an LNA oligonucleotide specific for the plastid of the first group. The LNA oligonucleotides specific for the plastid in the remaining group will be investigated in a future study. The designed LNA oligonucleotides that competed with modified 63f and 1492r were named LNA-Pla63 and LNA-Pla1492, respectively.

Based on the alignment data, DNA bases specific for plant organelles were replaced by LNA bases, which were equally distributed throughout the sequences, except for the overlapped bases. Four to six DNA bases were replaced based on the consideration that the *Tm* values should have been 70°C, using the automatic calculator on the EXIQON website (https://www.exiqon.com/ls), and ordered over the Gene Design website (http://www.genedesign.co.jp/). The replaced bases were indicated with bold letters in Table 1, and the designed LNA oligonucleotides were summarized in Table 2. The 3′ ends of all the LNA oligonucleotides were phosphorylated to avoid their extension during PCR.

The Probe Match program was used to determine whether the designed LNA oligonucleotides matched the sequences of bacterial SSU rRNA genes. The LNA oligonucleotides LNAMit63, LNA-Mit1492, LNA-Pla63, and LNA-Pla1492 fully matched 1, 26, 1, and 3 bacterial gene sequences, respectively, in approximately 2.7 million of the registered sequences. The sequences were affiliated with *Proteobacteria*, *Actinobacteria*, *Firmicutes*, and *Acidobacteria*. However, because the percentages of the matched sequences were negligible in the total sequences, the designed LNA oligonucleotides were considered to be available for the LNA oligonucleotide– PCR clamping technique in order to selectively amplify the SSU rRNA genes of bacteria associated with the individual plant parts by PCR.

### Determination of effective concentrations of LNA oligonucleotides

The effective concentrations of the LNA oligonucleotides were examined using 0 μM, 0.5 μM, 1.0 μM, 2.0 μM, 3.0 μM, and 4.0 μM to the DNAs extracted from the respective plant samples. Aseptic roots were also used as controls to confirm the mobility position of the PCR products of the mitochondria and plastid genes in the agarose gel after electrophoresis. According to the registered DNA sequences in the databases, the mitochondrial and plastid genes generate 1616 bp (NC_011033) and 1416 bp (NC_001320) products in rice and 1876 bp (GU985444) and 1416 bp (NC_002762) products in wheat, respectively, after being amplified with the modified 63f and 1492r primer sets. On the other hand, the bacterial genes generate products of different sizes (about 1470 bp). These differences were approximately 200 bp and 400 bp for the mitochondria of rice and wheat and approximately 100 bp for the plastids of both plants. These differences indicated that the products could be visually distinguished by agarose gel electrophoresis.

**Fig. 2:**
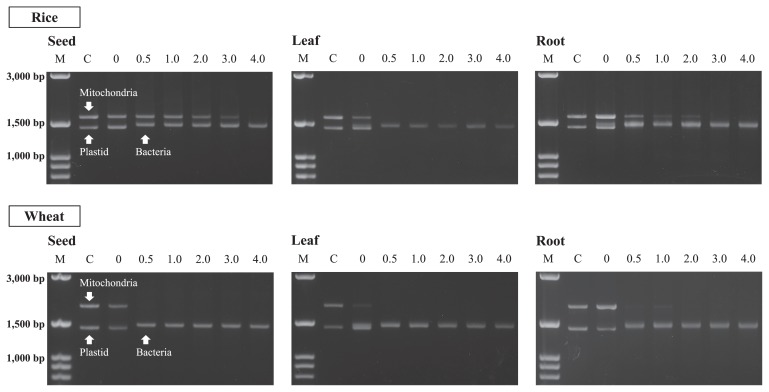
Agarose gel electrophoresis of nested PCR products derived from the different parts of rice and wheat plants to determine the effective concentrations of LNA oligonucleotides. The concentrations tested were 0, 0.5, 1.0, 2.0, 3.0, and 4.0 μM. “M” is the marker of 100 bp ladders and “C” is the control prepared from aseptic roots to generate mitochondria and plastid products.

Fig. 2 shows the amplification products of SSU rRNA genes from the seeds, leaves, and roots of rice and wheat plants, which were obtained using serial concentrations of LNA oligonucleotides. Mitochondria and plastid genes were dominantly observed in the products amplified without LNA oligonucleotides (0 μM), while no or the faint detection of bacterial genes was observed. However, when 0.5 μM LNA oligonucleotides were used, no products of the plastid genes were observed in any of the examined samples. No products of mitochondria genes from the rice leaf, wheat seed, or wheat leaf were also observed at the concentration. In the case of the rice seed, rice root, and wheat root, the intensities of the mitochondria products were significantly decreased when 0.5 μM LNA oligonucleotides were used, whereas bacterial products were observed with high intensities. The intensities of mitochondria products then decreased when LNA oligonucleotide concentrations were increased. The products were not detected at 4.0 μM, 3.0 μM, or 2.0 μM LNA oligonucleotides for the rice seed, rice root, and wheat root, respectively. This result indicated that LNA oligonucleotide– PCR clamping could inhibit the amplification of organelle genes, and 4.0 μM LNA-Mit (63 and 1492) and 0.5 μM LNA-Pla (63 and 1492) were effective concentrations for selectively amplifying the SSU rRNA genes of plant-associated bacteria, irrespective of the plant parts.

In our previous study (39), PNA-Mit1492 and PNA-Pla1492 were designed and used to examine the effects of the PNA PCR-clamping technique. Although the technique was useful for suppressing the amplification of mitochondria and plastid genes, the effect was insufficient because the amplicons of these genes were still detected with certain intensities, even when root samples were used. Therefore, application of the technique to seed and leaf samples, the plant organelle genes of which were represented more in the extracted DNA than bacterial genes, was considered to be difficult. However, LNA oligonucleotide–PCR clamping enabled selective amplification of the bacterial genes by applying PCR clamping to the forward primer side, while inhibiting the amplification of organelle genes.

### Comparison of DGGE patterns generated with and without LNA oligonucleotides

**Fig. 3:**
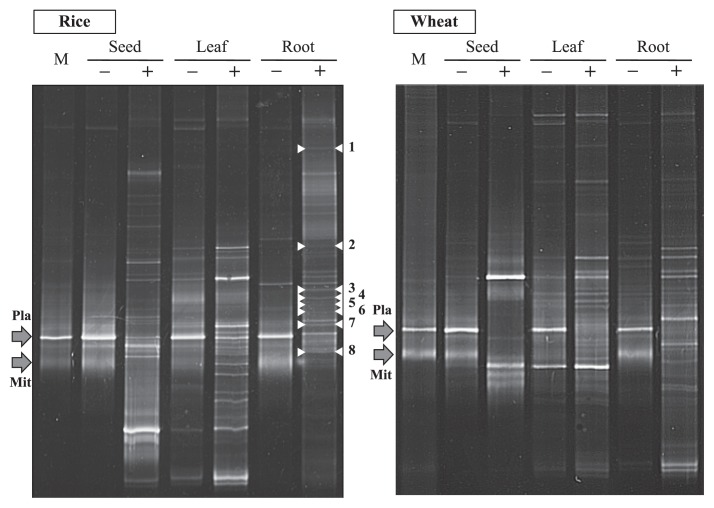
DGGE patterns of SSU rRNA genes derived from the different parts of rice and wheat plants. Symbols “−” and “+” indicate the lanes prepared without and with LNA oligonucleotides. “M” is the marker used to generate the DGGE bands of plastid and mitochondria, which are indicated as “Pla” and “Mit”, respectively. DGGE bands indicted with numbers 1 to 8 were sequenced to obtain the closest relatives using the DNA database.

Nested PCR products, which had been amplified with 341f-GC (30) and 907r (31), were used in the DGGE analysis conducted to examine the effects of LNA oligonucleotide– PCR clamping by comparing DGGE patterns. Products amplified without (0 μM) and with (4.0 μM) LNA oligonucleotides were used as DNA templates after purification. As shown in Fig. 3, the DGGE patterns generated without LNA oligonucleotides were similar to those generated from aseptic roots. Although some faint DGGE bands derived from bacterial SSU rRNA genes were also detected, two dominant bands showed the same mobility as those relative to mitochondria and plastids. This phenomenon has been attributed to the excessive amplification of organelle genes and has resulted in a critical drawback when investigating the community structures of plant-associated bacteria (6, 12, 13, 27, 33, 40, 49). This was particularly difficult in samples with a low bacterial DNA/plant organelle DNA ratio, and trials have been conducted to solve the associated problems (5, 11). However, when LNA oligonucleotides were used, bacterial DGGE bands that were hidden due to low amplification caused by the predominance of the ribosomal sequences of plant organelles significantly increased, even in seed samples, while no mitochondrial or plastid bands were detected.

Hardoim *et al.* (8) investigated the dynamics of seed-borne rice endophytes on the early plant growth stage using PCR-DGGE and sequencing analyses, and found that the nested PCR system (799F–1492R followed by 968F–1401R) (4) was the most efficient approach to detect rice endophytic bacteria by a PCR-DGGE analysis. However, the bacterial bands detected from rice seeds, shoots, and roots appeared to be less than those observed in the present study. The primer set used for nested PCR exhibited low coverage (56.6% for 968f and 62.0% for 1401r). In contrast, the LNA oligonucleotide–PCR clamping technique has the advantage of amplifying almost full-length SSU rRNA genes using primer sets with higher coverage. Since the upper half of the sequence of bacterial SSU rRNA genes is known to be more informative than the latter half (21, 50) due to its higher divergence, this technique was considered effective and useful for investigating the community structures of plant-associated bacteria irrespective of the parts.

### Closest relatives of DGGE bands newly detected using LNA oligonucleotides

DGGE bands newly detected in rice roots by LNA oligonucleotides were sequenced, and the closest relatives are listed in Table 3. As shown in Table 3, similarities with the known isolated bacteria were less than 97%. Stackebrandt and Goebel (42) suggested that a SSU rRNA gene sequence similarity of 97% should become the boundary for the delineation of prokaryotic species, and this has been accepted among microbiologists. Stackebrandt and Ebers (43) then proposed a more relaxed cut-off of 98.7–99%, after inspecting a large amount of recently published findings. Although we determined partial sequences in the upper half of the sequences of bacterial SSU rRNA genes, some of the closest relatives showed lower similarities, ranging from the lower 90% to the higher 80% levels.

*Agromyces*, *Bacillus*, *Caulobacter*, *Flavobacterium*, *Frigoribacterium*, *Kucuria*, *Methylobacterium*, *Microbacterium*, *Micrococcus*, *Ochlobacterium*, *Panenibacillus*, *Pantoea*, *Plantibacter*, *Pseudomonas*, *Sphingomonas*, and *Stenotrophomonas* have been isolated from the seeds and seedlings of rice plants by culture-dependent cultivation techniques (8, 16, 36). *Achromobacter*, *Acinetobacter*, *Aeromonas*, *Azoarcus*, *Bacillus*, *Enterobacter*, *Escherichia*, *Deinococcus*, *Dyella*, *Herbaspirillum*, *Janthinobacterium*, *Neisseria*, *Pantoea*, *Pseudomonas*, *Rhizobium*, and *Stenotrophomonas* have been obtained by culture-independent molecular techniques (8, 13). Although these bacteria were not identified as the closest relatives, this result suggested the potential of this technique to provide an insight into detecting novel bacteria that were not identified in previous studies, presumably because they were hidden by low amplification due to the predominant ribosomal sequences of plant organelles

In conclusion, the LNA oligonucleotide–PCR clamping technique enabled the detection of bacterial genes that were hidden by low amplification due to the predominant ribosomal sequences of plant organelles. In addition, the closest relatives of DGGE bands newly detected by LNA oligonucleotides showed low similarity with known bacteria, which suggested that novel bacteria may not have been detected in previous studies. Thus, the progressive technique developed in this study has the potential to become a breakthrough in solving the critical drawback associated with investigating the community structures of plant-associated bacteria in order to enhance understanding of the roles of bacteria in plant growth.

## Supplementary Information



## Figures and Tables

**Table 1:**
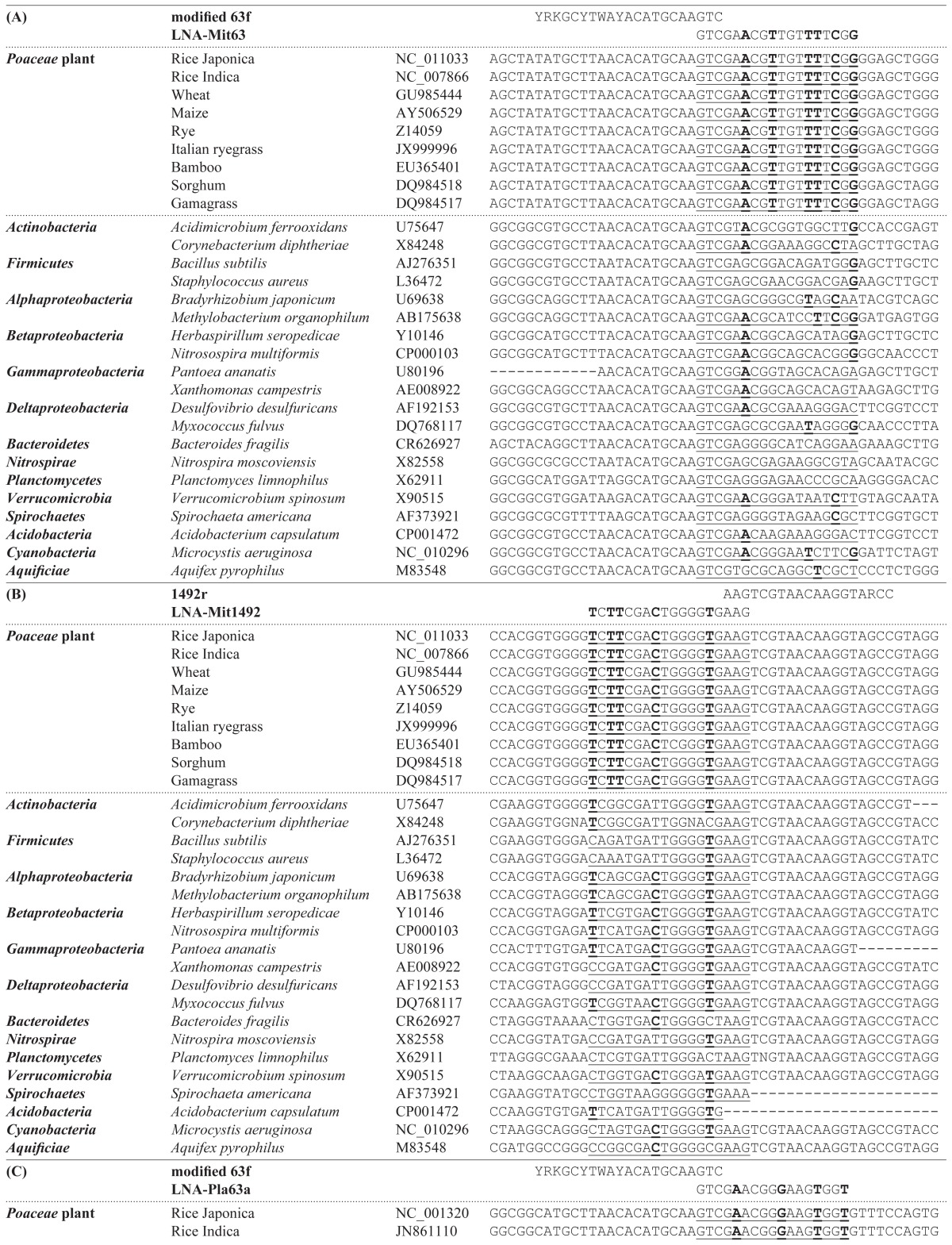

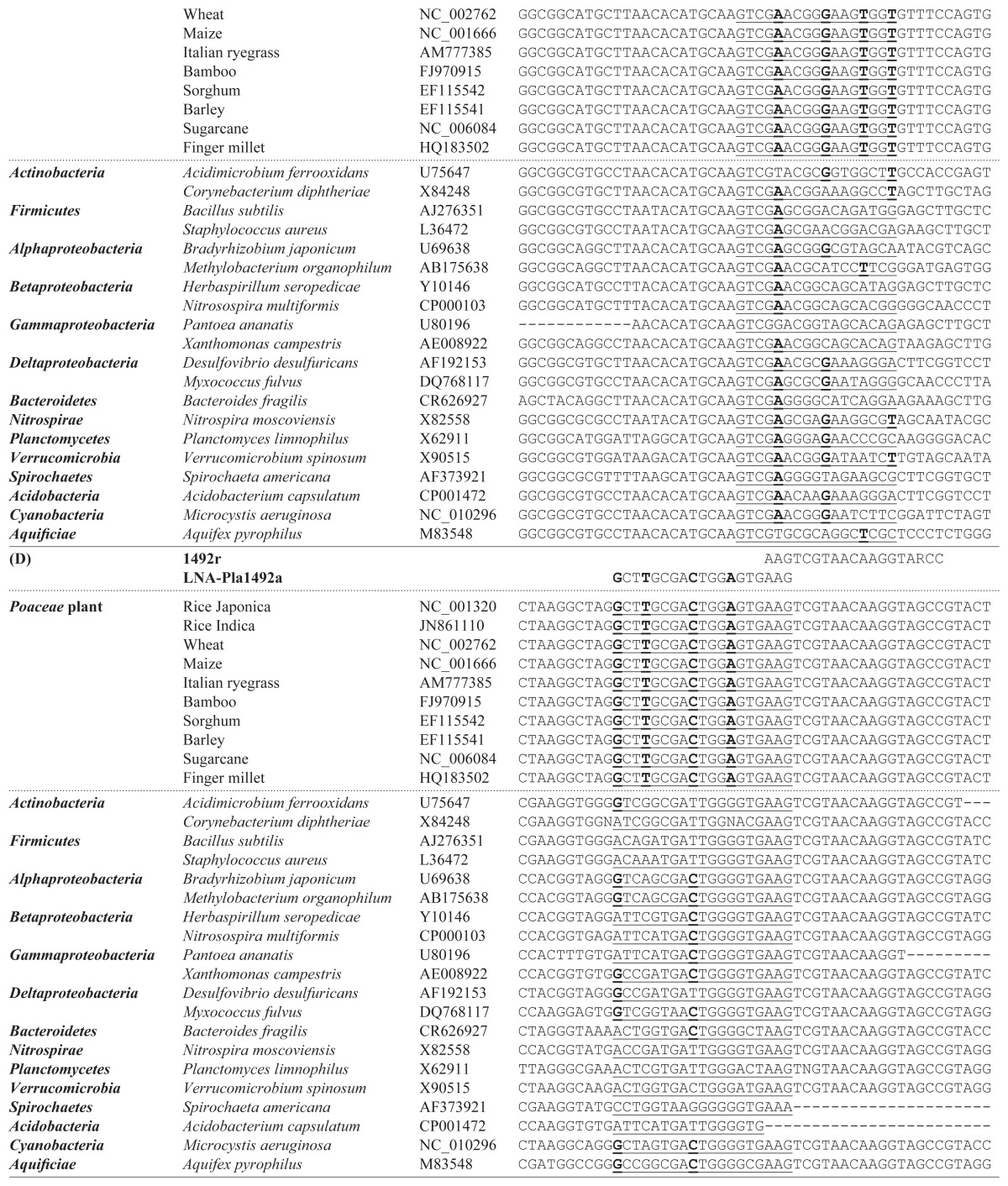
Alignment of bacterial primer sequences with the corresponding region of the SSU rRNA genes from plant organelle (either mitochondria or plastids) for the design of LNA oligonucleotides

1A and 1B show that the design drawings of LNA oligonucleotides competed with the modified 63f primer and 1492r primer at the mitochondria genes, respectively. 1C and 1D show the design drawings of LNA oligonucleotides that competed with the modified 63f primer and 1492r primer at the plastid genes, respectively. Underlined sequences correspond to the sequences of the designed LNA oligonucleotides. Bold letters indicate the LNA bases that replaced the DNA bases.

**Table 2:** Sequences of the designed LNA oligonucleotides specific for the SSU rRNA genes from plant mitochondria and plastids

Name of the LNA oligonucleotide	Sequence	Length (bases)	*Tm* (°C)
LNA-Mit63	5′-GTCGA**A**CG**T**TGT**TT**T**C**G**G**p-3′	18	70
LNA-Mit1492	5′-CTTC**A**CCCCA**G**TCG**AA**G**A**p-3′	18	70
LNA-Pla63	5′-GTCG**A**ACGG**G**AAG**T**GG**T**p-3′	17	70
LNA-Pla1492	5′-CTTCAC**T**CCA**G**TCGC**A**AG**C**p-3′	19	70

LNA bases are indicated with bold letters. Underlined DNA bases are overlapped sequences with the bacterial primer. The 3′ end of all LNA were phosphorylated to avoid extension during PCR. This was indicated with a small letter “p”.

**Table 3:** Closest relatives of the DGGE bands of bacterial SSU rRNA genes derived from rice roots, the bands of which were additionally detected using LNA oligonucleotides

DGGE band	Closest relatives	Accession number	Similarity	Seq (bp)	Alignment
1	Uncultured *Sphingobacteria* bacterium clone GASP-WC2W1	EF075155	99%	449	447/449
	*Terrimonas lutea* DY	NR_041250	96%	449	434/453

2	Uncultured *Bacteroidetes* bacterium clone SNNP_2012-140	JX114473	92%	546	500/546
	*Bacteroidetes* bacterium THS	AB539999	83%	546	460/552

3	Uncultured bacterium clone Amb_16S_723	EF018351	99%	483	480/483
	*Solibacter usitatus* Ellin6076	CP000473	95%	483	460/484

4	Uncultured bacterium clone 0693	JN855457	95%	540	512/538
	*Chryseolinea serpens* RYG	FR774778	92%	540	498/540

5	Uncultured bacterium clone 1142	JN855318	99%	540	537/538
	*Chryseolinea serpens* RYG	FR774778	93%	540	503/541

6	Uncultured bacterium clone OTU98	JN222519	88%	549	487/551
	*Desulfuromonas* sp. TZ1	JX258673	84%	549	468/555

7	Uncultured bacterium clone BSS126	HQ397574	95%	544	518/545
	*Bacillus megaterium* S9	JQ410775	93%	544	504/543

8	Uncultured *Acidobacteria* bacterium clone GASP-WC2W1_D09	EF075181	98%	531	522/531
	*Telmatobacter* sp. 15-28	KC954751	83%	531	445/537
