# Supplementary material for: Application of Locked Nucleic Acid (LNA) Oligonucleotide–PCR Clamping Technique to Selectively PCR Amplify the SSU rRNA Genes of Bacteria in Investigating the Plant-Associated Community Structures
Source: Microbes Environ. 2014 Jul 17;29(3):286–95. doi: 10.1264/jsme2.ME14061 (PMC4159040; doi:10.1264/jsme2.ME14061)
Supplement: Supplementary file 1 [file 29_286_s1.pdf]

Table S1 List of the SSU rRNA genes from plant mitochondria used for design of LNA oligonucleotides that competed with modified 63f and 1492r. Symbol ○ indicates the genes that were identical with designed LNA oligonicleotides, while symbol ▲ indicates the genes that showed mismatches in the concerned regions with designed LNA oligonucleotide. The – indicates that the corresponding sequence were not registered in DNA database. The N.D. indicates not determined.

Family	Binomial name	Common name	Accession number	LNA-Mit63	LNA-Mit1492
Acoraceae	*Acorus gramineus*	Japanese rush	DQ008668	–	○
Adoxaceae	*Sambucus canadensis*	American elderberry	AF194000	○	○
Alismataceae	*Alisma plantago-aquatica*	Northern water plantain	DQ008669	○	–
Amaranthaceae	*Beta macrocarpa*	Beet	FQ378026	▲	○
Amborellaceae	*Amborella trichopoda*	N.D.	AF193987	○	○
Annonaceae	*Annona muricata*	Soursop	DQ008670	○	○
	Polyalthia suberosa	N.D.	AF193991	○	○
Apiaceae	*Daucus carota*	Carrot	JQ248574	○	○
Apodanthaceae	*Pilostyles thurberi*	Thurber's stemsucker	U82655	○	–
Araceae	*Orontium aquaticum*	Golden club	DQ008672	–	○
	Spathiphyllum clevelandii	Peace lily	AF193975	○	–
	Spathiphyllum wallisii	Peace lily	DQ008673	○	○
	Amorphophallus konjac	Konjac	N.D.	–	–
Araliaceae	*Panax ginseng*	Korean ginseng	EF550996	○	○
Araucariaceae	*Agathis borneensis*	borneo kauri	AB029363	–	○
	Araucaria excelsa	Norfolk island pine	AB029364	–	○
Arecaceae	*Phoenix dactylifera*	Date Palm	JN375330	○	○
Aristolochiaceae	*Aristolochia macrophylla*	Dutchman's pipe	DQ008674	▲	○
	Asarum canadense	Canada wild ginger	DQ008676	▲	○
	Saruma henryi	Upright wild ginger	DQ008677	○	○
Aroideae	*Xanthosoma mafaffa*	Giant golden taro	AF193974	○	○
Asparagaceae	*Yucca filamentosa*	Adam's needle	U82643	○	–
Aspleniaceae	*Asplenium nidus*	Nest fern	AB029354	○	–
Asteraceae	*Lactuca sativa*	Lettuce	HM047292	○	–
	Helianthus annuus	Sunflower	N.D.	–	–
Atherospermataceae	*Atherosperma moschatum*	Southern sassafras	DQ008679	○	○
Athyriaceae	*Diplazium pycnocarpon*	Narrow leaved glade fern	AF058662	○	–
Balanophoraceae	*Helosis cayennensis*	N.D.	U82640	○	–
Berberidaceae	*Podophyllum peltatum*	Mayapple	DQ008683	○	○
Brassicaceae	*Arabidopsis thaliana*	Thale cress	JF729200	▲	○
	Brassica campestris	Biennial turnip rape	NC_016125	▲	○
	Brassica carinata	Ethiopian mustard	JF920287	▲	○
	Brassica juncea	Mustard greens	JF920288	▲	○
	Brassica napus	Rape	FR715249	▲	○
	Brassica oleracea	Cabbage	NC_016118	▲	○
	Brassica oleracea	Wild cabbage	JF920286	▲	○
	Brassica rapa	Pak choi	JF920285	▲	○
	Raphanus sativus	Radish	AB694744	▲	○
	Brassica pekinensis	Chinese cabbage	N.D.	–	–
	Raphanus sativus	Radish	N.D.	–	–
Butomaceae	*Butomus umbellatus*	Flowering rush	KC208619	○	▲
Buxaceae	*Buxus* sp.	Box	AF193996	○	○
	Didymeles perrieri	N.D.	DQ008696	–	○
Cabombaceae	*Cabomba sp.*	N.D.	AF193982	○	○
Calycanthaceae	*Calycanthus floridus*	Eastern sweetshrub	AF193989	○	▲
Canellaceae	*Canella winterana*	Cinnamon bark	DQ008687	○	○
	Cinnamodendron ekmanii	N.D.	DQ008688	○	○
Cannabaceae	*Humulus lupulus*	Hop	N.D.	–	–
Caricaceae	*Carica papaya*	Papaya	EU431224	○	○
Caryophyllaceae	*Silene latifolia*	White campion	HM562727	▲	○
	Silene noctiflora	Night flowering catchfly	JF750462	▲	○
	Silene vulgaris	Bladder campion	HM562728	▲	○
Celastraceae	*Maytenus hookeri*	N.D.	AY855839	–	○
Cephalotaxaceae	*Cephalotaxus harringtonia*	N.D.	AB029365	▲	○
Ceratophyllaceae	*Ceratophyllum demersum*	Hornwort	AF193977	○	▲
Chenopodiaceae	*Beta vulgaris*	Sugar beet	NC_002511	▲	○
	Spinacia oleracea	Spinach	N.D.	–	–
Chloranthaceae	*Chloranthus multistachys*	Multi stacked chloranthus	DQ008692	○	○
	Hedyosmum arborescens	N.D.	DQ008693	○	○
	Sarcandra grandifolia	N.D.	AF193992	○	○
Clusiaceae	*Hypericum calycinum*	Rose of sharon	U82651	○	–

Convolvulaceae	*Ipomoea batatas*	Sweet potato	N.D.	–	–
Crossosomataceae	*Crossosoma bigelovii*	Ragged rockflower	AF194001	○	○
Cucurbitaceae	*Citrullus lanatus*	Watermelon	NC_014043	○	○
	Cucumis africanus	Wild cucumber	AY357205	○	–
	Cucumis melo	Melon	JF412792	○	○
	Cucumis sativa	Cucumber	NC_016005	▲	○
	Cucumis zeyherii	South African spiny cucumber	AY357207	○	–
	Cucurbita moschata	Winter squash	AY357208	–	○
	Cucurbita pepo	Summer squash	AY357209	–	○
	Cucurbita moschata	Pumpkin	N.D.	–	–
	Luffa cylindrical	Loofah	N.D.	–	–
Cupressaceae	*Callitropsis nootkatensis*	Nootka cypress	AB029366	▲	○
	Cryptomeria japonica	Japanese cedar	AB029367	▲	○
	Juniperus chinensis	Chinese juniper	AB029368	▲	○
Cycadaceae	*Cycas taitungensis*	Prince sago	AP009381	▲	○
Cyclanthaceae	*Carludovica palmata*	Panama hat plant	DQ008694	○	○
Cytinaceae	*Bdallophyton americanum*	N.D.	U82653	○	–
	Cytinus ruber	N.D.	U82639	○	–
Degeneriaceae	*Degeneria vitiensis*	N.D.	DQ008695	○	○
Equisetaceae	*Equisetum arvense*	Field horsetail	AF058663	○	–
Euphorbiaceae	*Jatropha curcas*	Barbados nut	GQ386927	○	–
	Ricinus communis	Castor bean	HQ874649	○	○
	Manihot utilissima	Cassava	N.D.	–	–
Eupomatiaceae	*Eupomatia bennettii*	Small bolwarra	DQ008698	○	○
Eupteleaceae	*Euptelea polyandra*	N.D.	AF193997	○	○
Fabaceae	*Cajanus cajan*	Gandule bean	EU307415	○	–
Fabaceae	*Glycine max*	Soybean	M16859	○	○
	Lotus japonicus	N.D.	JN872551	○	▲
	Lupinus luteus	European yellow lupine	Z11512	○	○
	Millettia pinnata	Indian beech	JN872550	○	○
	Phaseolus coccineus	Runner bean	EU307418	○	–
	Phaseolus vulgaris	Common bean	EU307370	○	–
	Pisum sativum	Pea	X98799	○	–
	Vicia faba	Broad bean	KC189947	○	○
	Vigna angularis	Azuki bean	AP012599	○	○
	Vigna radiata	Mung bean	HM367685	○	○
	Vigna unguiculata	Cowpea	EU307376	○	–
	Medicago sativa	Alfalfa	N.D.	–	–
Geraniaceae	*Geranium himalayense*	Lilac cranesbill	AF194003	○	○
	Geranium macrorrhizum	Bigroot geranium	DQ317028	○	○
	Hypseocharis pimpinellifolia	N.D.	AF194002	○	○
	Monsonia emarginata	Mountain geranium	DQ317029	○	▲
	Erodium pelargoniflorum	N.D.	DQ317006	○	○
	Erodium trifolium	N.D.	DQ317007	○	○
	Sarcocaulon vanderietiae	N.D.	DQ317004	○	○
Gesneriaceae	*Boea hygrometrica*	N.D.	JN107812	▲	○
Hernandiaceae	*Gyrocarpus americanus*	Helicopter tree	DQ008702	▲	○
Himantandraceae	*Galbulimima belgraveana*	White magnolia	DQ008704	○	○
Hydnoraceae	*Hydnora africana*	Jackal's kost	U82637	○	–
Lardizabalaceae	*Akebia quinata*	Chocolate vine	DQ008709	○	○
	Lardizabala biternata	Lardizabala	DQ008710	○	○
	Sargentodoxa cuneata	N.D.	DQ008729	–	○
Lauraceae	*Cinnamomum camphora*	Camphor Laurel	DQ008711	○	○
	Lindera benzoin	Wild allspice	U82646	○	○
Lemnoideae	*Spirodela polyrhiza*	Great duckweed	JQ804980	○	○
Liliaceae	*Pleea tenuifolia*	N.D.	DQ008735	○	○
	Allium cepa	Onion	N.D.	–	–
	Allium fistulosum	Welsh onion	N.D.	–	–
	Allium sativum	Garlic	N.D.	–	–
	Asparagus officinalis	Asparagus	DQ008678	–	–
Magnoliaceae	*Liriodendron tulipifera*	Tulip tree	KC821969	○	○
	Magnolia grandiflora	Southern magnolia	AF161089	○	○
	Magnolia × soulangeana	Saucer magnolia	U82648	○	–
Malvaceae	*Gossypium hirsutum*	Cotton	N.D.	–	–
	Hibiscus esculentus	Gumbo	N.D.	–	–
Menispermaceae	*Cocculus trilobus*	N.D.	DQ008714	○	○

Monimiaceae	*Hedycarya arborea*	Pigeonwood	DQ008716	–	○
	Hortonia floribunda	N.D.	DQ008717	–	○
Myristicaceae	*Myristica fragrans*	Nutmeg	DQ008718	▲	○
Nelumbonaceae	*Nelumbo nucifera*	Lotus	AF193983	○	▲
Nymphaeaceae	*Nuphar* sp.	Pond lily	AF193981	○	▲
	Nymphaea sp.	Water lilies	AF161091	○	○
	Euryale sp.	N.D.	AF193980	○	▲
	Victoria cf. *amazonica*	N.D.	AF193979	○	○
Onagraceae	*Oenothera berteriana*	Bertero's evening primrose	X61277	○	○
Orchidaceae	*Dendrobium officinale*	N.D.	KC465231	▲	○
	Neottia nidus-avis	Bird's nest Orchid	U82644	○	–
Papaveraceae	*Sanguinaria canadensis*	Bloodwort	DQ008720	○	○
	Dicentra sp.	N.D.	DQ008700	○	○
Pedaliaceae	*Sesamum indicum*	Sesame	N.D.	–	–
Pinaceae	*Abies homolepis*	Nikko fir	AB029360	–	○
	Pinus strobus	Eastern white pine	AF058659	–	○
Piperaceae	*Peperomia obtusifolia*	Baby rubber plant	DQ008722	▲	○
	Piper betle	Betel	AF161088	▲	○
	Peperomia obtusifolia	Baby rubber plant	DQ008722	▲	○
	Piper betle	Betel	AF161088	▲	○
	Peperomia fosterii	N.D.	AF193986	▲	○
	Piper nigrum	Pepper	N.D.	–	–
Plantaginaceae	*Plantago atrata*	N.D.	AJ389618	–	○
	Plantago australis	N.D.	AY818949	–	○
	Plantago coronopus	Minutina	AJ389617	–	○
	Plantago cyanops	N.D.	AJ389620	–	○
	Plantago lanceolata	Ribwort plantain	AJ389619	–	○
	Plantago media	Hoary plantain	AJ389614	–	○
	Plantago rigida	N.D.	AJ389616	–	○
	Plantago sericea	N.D.	AJ389621	–	○
Platanaceae	*Platanus occidentalis*	American sycamore	U82650	○	–
Poaceae	*Aegilops ventricosa*	Goatgrass	DQ307262	○	–
	Bambusa oldhamii	Bamboo	EU365401	○	○
	Eleusine coracana	Finger millet	KC119749	–	○
	Eremopyrum bonaepartis	N.D.	DQ307271	○	–
	Lolium perenne	Perennial ryegrass	JX999996	○	○
	Oryza rufipogon	Brownbeard rice	AP011076	○	○
	Oryza sativa Indica	Indica rice	NC_007866	○	○
	Oryza sativa Japonica	Japonica rice	NC_011033	○	○
	Psathyrostachys juncea	Russian wildrye	DQ307265	○	–
	Secale cereale	Rye	Z14059	○	○
	Secale strictum	Mountain rye	DQ307259	○	–
	Secale sylvestre	N.D.	DQ307272	○	–
	Sorghum bicolor	Sorghum	DQ984518	○	○
	Thinopyrum bessarabicum	Wheatgrass	DQ307269	○	–
	Thinopyrum intermedium	Intermediate wheatgrass	DQ677514	○	–
	Tripsacum dactyloides	Gamagrass	DQ984517	○	○
	Triticum aestivum	Wheat	GU985444	○	○
	Zea luxurians	Guatemalan teosinte	DQ645537	○	○
	Zea mays	Maize	AY506529	○	○
	Zea perennis	Perennial teosinte	DQ645538	○	○
	Crithopsis delileana	N.D.	DQ307273	○	–
	Dasypyrum breviaristatum	N.D.	GU168590	○	–
	Ferrocalamus rimosivaginus	N.D.	JQ235168	○	○
	Haynaldia villosa	N.D.	DQ307266	○	–
	Lolium temulentum	Darnel	N.D.	–	–
	Hordeum vulgare	Barley	N.D.	–	–
	Ragmites australis	Reed	N.D.	–	–
	Saccharum officinarum	Sugarcane	N.D.	–	–
	Avena sativa	Oats	N.D.	–	–
Podocarpaceae	*Podocarpus costalis*	Arius	AB029369	▲	○
	Nageia nagi	N.D.	AB029361	▲	○
Polygonaceae	*Fagopyrum esculentum*	Buckwheat	N.D.	–	–
Polypodiaceae	*Polypodium aureum*	Golden polypody	AF058665	○	–
Proteaceae	*Grevillea robusta*	Silky oak	AF193995	▲	○
Pteridaceae	*Adiantum pedatum*	Northern maidenhair fern	AF058660	○	–

Rafflesiaceae	*Rafflesia pricei*	Rafflesia	U96694	○	○
Ranunculaceae	*Ranunculus* sp.	Buttercup	AF161093	○	–
Ranunculoideae	*Clematis* sp.	traveller's joy	AF193994	▲	○
Rosaceae	*Malus domestica*	Apple	FR714868	○	○
	Fragaria x ananassa	Strawberry	N.D.	–	–
Rutaceae	*Citrus maxima*	Pomelo	FJ356261	–	○
Sabiaceae	*Meliosma squamulata*	N.D.	DQ008728	○	–
	Sabia sp.	N.D.	DQ008727	○	○
Santalaceae	*Lepidoceras chilense*	N.D.	U82641	▲	○
Schisandraceae	*Kadsura japonica*	Kadsura	AF193985	○	○
	Schisandra sphenanthera	five flavor berry	AF193984	▲	○
Sciadopityaceae	*Sciadopitys verticillata*	Japanese umbrella pine	AF161085	▲	○
Scrophulariaceae	*Mimulus guttatus*	Monkey flower	JN098455	▲	○
Siparunaceae	*Siparuna decipiens*	N.D.	DQ008733	○	▲
Smilacaceae	*Smilax hispida*	N.D.	U82645	○	–
Solanaceae	*Nicotiana tabacum*	Tobacco	BA000042	○	○
	Solanum tuberosum	Potato	AJ252732	○	–
	Solanum melongena	Eggplant	N.D.	–	–
	Lycopersicum esculentum	Tomato	N.D.	–	–
Stemonaceae	*Croomia pauciflora*	N.D.	DQ008734	○	○
Taxaceae	*Taxus brevifolia*	Pacific yew	AB029370	▲	○
Thelypteridaceae	*Phegopteris hexagonoptera*	Broad beech fern	AF058666	○	▲
Tiliaceae	*Corchorus capsularis*	Jute	N.D.	–	–
Tofieldiaceae	*Tofieldia calyculata*	N.D.	DQ008736	○	○
Trochodendraceae	*Tetracentron sinense*	Spur leaf	AF193998	○	○
	Trochodendron aralioides	Wheel tree	AF161092	○	○
Vitaceae	*Vitis vinifera*	Grape	FM179380	○	○
Winteraceae	*Drimys winteri*	Winter's bark	AF197162	▲	○
	Takhtajania perrieri	N.D.	DQ008740	○	○
	Tasmannia insipida	Brush pepperbush	DQ008739	▲	○
Zamiaceae	*Dioon edule*	Palma de la Virgen	AF058657	▲	○
Zingiberaceae	*Zingiber officinale*	Ginger	N.D.	–	–

Table S2 List of the SSU rRNA genes from plant plastid used for design of LNA oligonucleotides that competed with modified 63f and 1492r. Symbol ○ indicates the genes that were identical with designed LNA oligonicleotides, while symbol ● indicates the genes that were identical with the other plant groups. Symbol ▲ indicates the genes that showed mismatches in the concerned regions with both plant groups. The – indicates that the corresponding sequence were not registered in DNA database. The N.D. indicates not determined.

Family	Binomial name	Common name	Accession number	LNA-Pla63	LNA-Pla1492
Acoraceae	*Acorus calamus*	Sweetflag	AJ879453	●	●
Amaranthaceae	*Beta vulgaris*	Sugar beet	EF534108	●	●
	Spinacia oleracea	Spinach	NC_002202	●	●
Amborellaceae	*Amborella trichopoda*	N.D.	AJ506156	●	●
Apiaceae	*Daucus carota*	Carrot	NC_008325	●	●
Araceae	*Lemna minor*	Common duckweed	DQ400350	●	●
Asparagaceae	*Asparagus officinalis*	Garden asparagus	HQ183492	●	●
Asteraceae	*Helianthus annuus*	Common sunflower	NC_007977	●	●
	Lactuca sativa	Garden lettuce	NC_007578	●	●
Berberidaceae	*Nandina domestica*	Heavenly bamboo	DQ923117	●	●
Brassicaceae	*Aethionema cordifolium*	Lebanon stonecress	AP009366	●	●
	Arabidopsis thaliana	Mouse-ear cress	AP000423	●	●
	Arabis alpina	Alpine rock-cress	HF934132	○	●
	Arabis hirsuta	Hairy rock-cress	AP009369	○	●
	Barbarea verna	Land cress	AP009370	●	●
	Brassica rapa	Chinese cabbage	NC_015139	●	●
	Draba nemorosa	Woodland whitlowgrass	AP009373	○	●
	Lepidium virginicum	Virginia pepperweed	AP009374	●	●
	Lobularia maritima	Sweet alyssum	AP009375	●	●
Buxaceae	*Buxus microphylla*	Littleleaf boxwood	EF380351	●	●
Calycanthaceae	*Calycanthus floridus*	Eastern sweetshrub	AJ428413	●	●
Campanulaceae	*Trachelium caeruleum*	Blue throatwort	EU090187	●	●
Caricaceae	*Carica papaya*	Papaya	EU431223	●	●
Ceratophyllaceae	*Ceratophyllum demersum*	Rigid hornwort	EF614270	●	●
Chloranthaceae	*Chloranthus spicatus*	Chulantree	EF380352	●	●
Convolvulaceae	*Cuscuta campestris*	Field dodder	AJ401353	○	●
	Cuscuta exaltata	Tall dodder	EU189132	▲	●
	Cuscuta gronovii	Common dodder	AM711639	○	●
	Cuscuta obtusiflora	Peruvian dodder	EU189133	○	●
	Cuscuta odorata	N.D.	AJ278624	○	●
	Cuscuta platyloba	N.D.	AJ401355	○	●
	Cuscuta reflexa	Southern Asian dodder	AM711640	▲	●
	Cuscuta subinclusa	N.D.	AJ278625	○	●
	Ipomoea purpurea	Common morning glory	EU118126	●	●
Cucurbitaceae	*Cucumis melo*	Muskmelon	NC_015983	●	●
	Cucumis sativus	Cucumber	NC_007144	●	●
Cupressaceae	*Calocedrus formosana*	N.D.	AB831010	○	●
	Calocedrus macrolepis	N.D.	EU161517	○	▲
	Chamaecyparis pisifera	Sawara false-cypress	EU161522	○	▲
	Cryptomeria fortunei	N.D.	DQ478788	○	●
	Cryptomeria japonica	Japanese cedar	AP009377	○	●
	Cupressus gigantea	N.D.	EU161525	○	▲
	Fokienia hodginsii	N.D.	EU161531	○	▲
	Glyptostrobus pensilis	N.D.	EU161532	○	▲
	Juniperus virginiana	Eastern redcedar	U24586	○	●
	Libocedrus sp.	N.D.	EU161538	○	●
	Microbiota decussata	N.D.	EU161541	○	●
	Platycladus orientalis	N.D.	EU161549	○	●
	Taxodium mucronatum	N.D.	EU161559	○	▲
	Tetraclinis articulata	N.D.	EU161560	○	●
	Thujopsis dolabrata	Hiba arborvitae	EU161562	○	●
Cycadaceae	*Cycas taitungensis*	Prince sago	AP009339	●	●
Dalbergieae	*Arachis hypogaea*	Peanut	EU982406	○	–
Dioscoreaceae	*Dioscorea elephantipes*	Elephant's foot	EF380353	●	●
Ecdeiocoleaceae	*Ecdeiocolea monostachya*	N.D.	HQ183501	○	▲
	Georgeantha hexandra	N.D.	HQ183505	●	○

Ephedraceae	*Ephedra distachya*	Joint-fir	EU161530	○	▲
	Ephedra equisetina	Ma huang	AP010819	○	▲
	Ephedra trifurca	Mexican-tea	U24584	○	▲
Euphorbiaceae	*Manihot utilissima*	Cassava	NC_010433	●	●
Fabaceae	*Glycine max*	Soybean	NC_007942	●	●
	Lotus japonicus	N.D.	AP002983	●	●
	Medicago sativa	Alfalfa	JX185400	○	●
	Medicago truncatula	Barrel medic	AC093544	○	●
	Phaseolus vulgaris	Kidney bean	DQ886273	●	●
Geraniaceae	*Pelargonium* × *hortorum*	Common geranium	DQ897681	●	▲
Gnetaceae	*Gnetum leyboldii*	N.D.	AF244555	○	▲
	Gnetum montanum	N.D.	KC427271	○	▲
	Gnetum parvifolium	Small-leaved jointfir	AP009569	○	▲
Juncaceae	*Juncus effusus*	Soft rush	HQ183510	○	▲
Lamiaceae	*Glechoma hederacea*	Ground-ivy	DQ417652	●	○
Magnoliaceae	*Liriodendron tulipifera*	Tuliptree	DQ899947	●	●
Malvaceae	*Gossypium barbadense*	Sea-island cotton	AP009123	●	●
	Gossypium hirsutum	Upland cotton	NC_007944	●	●
Moraceae	*Morus indica*	Mulberry	DQ226511	●	●
Myristicaceae	*Myristica yunnanensis*	N.D.	DQ629588	○	●
Nelumbonaceae	*Nelumbo nucifera*	Sacred lotus	NC_015610	●	●
Nymphaeaceae	*Nymphaea alba*	Castalia alba	AJ627251	●	●
Oleaceae	*Jasminum nudiflorum*	Winter jasmine	DQ673255	●	●
Onagraceae	*Oenothera argillicola*	Appalachian evening primrose	EU262887	●	▲
Orobanchaceae	*Epifagus virginiana*	Beechdrops	M81884	●	●
Passifloraceae	*Passiflora biflora*	Two-flowered passion flower	EU017103	○	▲
Pedaliaceae	*Sesamum indicum*	Oriental sesame	NC_016433	●	●
Pinaceae	*Pinus koraiensis*	Korean pine	AY228468	●	●
Piperaceae	*Piper cenocladum*	Ant piper	DQ887677	●	●
Platanaceae	*Platanus occidentalis*	Sycamore	DQ923116	●	●
Poaceae	*Acidosasa purpurea*	N.D.	HQ337793	○	○
	Aegilops cylindrica	Jointed goatgrass	KF534489	○	○
	Aegilops geniculata	N.D.	KF534490	○	○
	Aegilops speltoides	Goatgrass	AJ555401	○	○
	Aegilops tauschii	Tausch's goatgrass	AJ555402	○	○
	Agrostis capillaris	Colonial bentgrass	DQ417649	○	▲
	Agrostis stolonifera	Creeping bentgrass	EF115543	○	●
	Anomochloa marantoidea	Herbaceous bamboo	GQ329703	●	○
	Arundinaria gigantea	N.D.	JX235347	○	○
	Bambusa emeiensis	N.D.	HQ337797	○	○
	Bambusa oldhamii	Giant timber bamboo	FJ970915	○	○
	Brachypodium distachyon	Purple false brome	EU325680	○	○
	Chasmanthium latifolium	Wild oats	HM363119	○	○
	Coix lacryma-jobi	Job's tears	FJ261955	○	○
	Cryptochloa strictiflora	N.D.	JX235348	○	○
	Dendrocalamus latiflorus	Sweet giant bamboo	FJ970916	○	○
	Deschampsia antarctica	Antarctic hairgrass	KF887484	○	○
	Eleusine coracana	Indian finger millet	HQ183502	○	○
	Ferrocalamus rimosivaginus	N.D.	HQ337794	○	○
	Festuca altissima	Wood fescue	JX871939	○	○
	Festuca arundinacea	Tall fescue	FJ466687	○	○
	Festuca ovina	N.D.	JX871940	○	○
	Festuca pratensis	Lolium pratense	JX871941	○	○
	Hordeum vulgare	Barley	KC912687	○	○
	Indocalamus longiauritus	N.D.	HQ337795	○	○
	Leersia tisserantii	N.D.	JN415112	○	○
	Lolium multiflorum	Italian ryegrass	JX871942	○	○
	Lolium perenne	Perennial ryegrass	AM777385	○	○
	Microlaena stipoides	N.D.	GU592211	○	○
	Oryza australiensis	N.D.	GU592209	○	○
	Oryza meridionalis	N.D.	JN005831	○	○
	Oryza nivara	Indian wild rice	AP006728	○	○

	Oryza rufipogon	Brownbeard rice	KF428978	○	○
	Oryza sativa Indica	Indica rice	JN861109	○	○
	Oryza sativa Japonicum	Japonica rice	NC_001320	○	○
	Panicum virgatum	Switchgrass	HQ822121	○	○
	Phragmites australis	Common reed	KF730315	○	○
	Phyllostachys edulis	N.D.	HQ337796	○	○
	Phyllostachys nigra	Black bamboo	HQ154129	○	○
	Phyllostachys propinqua	N.D.	JN415113	○	○
	Potamophila parviflora	N.D.	GU592210	○	○
	Puelia olyriformis	N.D.	KC534841	○	○
	Rhynchoryza subulata	N.D.	JN415114	▲	○
	Saccharum hybrid	Sugarcane	AP006714	○	○
	Secale cereale	Rye	KC912691	○	○
	Setaria italica	Foxtail millet	KF646538	○	○
	Sorghum bicolor	Sorghum	EF115542	○	○
	Streptochaeta angustifolia	N.D.	HQ183525	●	○
	Triticum aestivum	Wheat	NC_002762	○	○
	Triticum monococcum	Einkorn wheat	KC912690	○	○
	Triticum turgidum	Poulard wheat	AJ555400	○	○
	Triticum urartu	Red wild einkorn	KC912693	○	○
	Zea mays	Maize	NC_001666	○	○
Polygonaceae	*Fagopyrum esculentum*	Common buckwheat	NC_010776	▲	●
Ranunculaceae	*Ranunculus macranthus*	Large buttercup	DQ359689	●	●
Rosaceae	*Fragaria × ananassa*	Strawberry	AY523534	●	–
Rubiaceae	*Coffea arabica*	Arabian coffee	EF044213	●	●
Rutaceae	*Citrus sinensis*	Sweet orange	DQ864733	●	●
Salicaceae	*Populus alba*	White poplar	AP008956	●	●
Schisandraceae	*Illicium oligandrum*	Star anise	EF380354	●	●
Solanaceae	*Solanum lycopersicum*	Tomato	NC_007898	▲	●
	Nicotiana tabacum	Common tobacco	NC_001879	●	●
Solaneae	*Solanum tuberosum*	Potato	NC_008096	▲	●
Taxaceae	*Austrotaxus spicata*	N.D.	EU161514	○	●
Vitaceae	*Vitis vinifera*	Grape	DQ424856	●	●
Welwitschiaceae	*Welwitschia mirabilis*	Tree tumbo	EU342371	○	▲
Winteraceae	*Drimys granadensis*	N.D.	DQ887676	●	●

Table S3 Numbers of bacterial SSU rRNA genes completely matched with the sequences of designed LNA oligonucleotides. Probe match program (http://rdp.cme.msu.edu/probematch/search.jsp) in the RDP11 was used for the search.

Phylogenetic groups in division levels	Total numbers of sequences	Number of matched sequences			
		Mit63	Mit1492	Pla63	Pla1492
Acidobacteria	69299	0	0	0	0
Actinobacteria	285047	0	1	2	0
Armatimonadetes	2916	0	0	0	0
Aquificae	2119	0	0	0	0
Bacteroidetes	335244	0	0	0	0
Caldiserica	364	0	0	0	0
Chlamydiae	1192	0	0	0	0
Chlorobi	3327	0	0	0	0
Chloroflexi	37285	0	0	0	0
Cyanobacteria	46045	0	0	0	0
Chrysiogenetes	13	0	0	0	0
Deferribacteres	1405	0	0	0	0
Deinococcus-Thermus	3899	0	0	0	0
Dictyoglomi	75	0	0	0	0
Elusimicrobia	415	0	0	0	0
Fibrobacteres	1054	0	0	0	0
Firmicutes	735125	1	1	0	1
Fusobacteria	14123	0	0	0	0
Gemmatimonadetes	4668	0	0	0	0
Lentisphaerae	2333	0	0	0	0
Nitrospira	7071	0	0	0	0
Planctomycetes	22171	0	0	0	0
Proteobacteria	878348	0	24	1	2
Spirochaetes	17349	0	0	0	0
Synergistetes	2695	0	0	0	0
Tenericutes	7732	0	0	0	0
Thermodesulfobacteria	241	0	0	0	0
Thermotogae	1030	0	0	0	0
Verrucomicrobia	24988	0	0	0	0
BRC1	599	0	0	0	0
OD1	876	0	0	0	0
OP11	305	0	0	0	0
SR1	967	0	0	0	0
TM7	4656	0	0	0	0
WS3	1068	0	0	0	0
Unclassified Bacteria	144584	0	0	0	0
**Total**	2660628	1	26	3	3
